# The impact of the implementation of hierarchical medical system on population health: evidence from China

**DOI:** 10.3389/fpubh.2024.1402832

**Published:** 2024-05-23

**Authors:** Ying Wang, Hong Chen

**Affiliations:** ^1^School of Public Administration and Law, Hunan Agricultural University, Changsha, Hunan, China; ^2^School of Humanities and Management, Hunan University of Chinese Medicine, Changsha, Hunan, China

**Keywords:** hierarchical medical system, population health, self-assessed health, chronic disease status, primary care

## Abstract

**Introduction:**

The implementation of a hierarchical medical system holds significant practical importance in advancing the Healthy China strategy and elevating the overall health status of the population of China.

**Methods:**

This article empirically examines the influence of the implementation of a hierarchical medical system on the health of the population using the latest 2020 China Family Panel Studies database. Furthermore, it investigates the variability of this impact across distinct health insurance participation statuses and literacy groups.

**Results:**

The findings of the study demonstrate that the implementation of the hierarchical medical system substantially enhances the health of the population, not only in terms of self-assessed health but also through a notable positive effect on alleviating chronic diseases. These results maintain their validity even after conducting robustness tests utilizing a replacement estimation model. Heterogeneity analysis reveals that the impact of the hierarchical medical system on the population’s health status exhibits significant variation concerning health insurance participation and literacy. Specifically, regarding health insurance participation, the hierarchical medical system effectively improves both self-assessed health and chronic disease status among the insured population. However, for those not enrolled in health insurance, the hierarchical medical system only demonstrates improvement in chronic disease status, with insignificant results observed in enhancing self-assessed health status. Moreover, propensity score matching (PSM) was also used to address endogeneity problems resulting from sample selectivity bias. The findings demonstrate that endogeneity issues can be suitably addressed by the PSM model. Additionally, they point out that an overestimation of the impact of the hierarchical medical system on the population’s self-assessed health state would result from failing to take sample selectivity bias into account. On the other hand, it will lead to the underestimation of the effect of the hierarchical medical system on the status of chronic diseases.

**Discussion:**

Moving forward, steadfast efforts should be directed toward further enhancing the implementation of the hierarchical medical system. This includes the comprehensive promotion and using the pivotal role of the hierarchical medical system in improving the health of the population.

## 1 Introduction

Health serves as a pivotal cornerstone for enhancing individuals’ well-being and upholding national social security and stability (1, 2). Since the establishment of the People’s Republic of China, the Communist Party of China (CPC) has prioritized the enhancement of population health, consistently placing the promotion of public health at the forefront of safeguarding and improving livelihoods (3–5). The introduction of the Healthy China Strategy in 2017, as outlined in the report of the 19th National Congress of the CPC, marked the formal elevation of population health promotion to the national strategic agenda. Furthermore, in the report of the 20th National Congress of the CPC in 2022, General Secretary Xi Jinping reiterated the imperative of “continuous promotion of the construction of a healthy China, with the priority of ensuring people’s health positioned strategically within development.” This reaffirms the enduring commitment of the party and the state to enhancing public health. The introduction of a hierarchical medical system was originally suggested in the 2006 “Guiding Opinions of The State Council of China on the Development of Urban Community Health Services, “which was a significant reform drive within the healthcare industry. This program launched community-based primary diagnostic pilots and introduced the idea of a two-way referral system and graded medical care. Subsequently, in 2015, China’s “Guiding Opinions on Promoting the Construction of Hierarchical Medical Systems” provided a comprehensive framework outlining the substance, fundamental principles, and developmental objectives of the graded diagnosis and treatment system. This delineated the formal commencement of hierarchical medical system construction in China. Initially conceived to attract the redistribution of high-quality medical resources and enhance grassroots population health (6), the core objective of the system is to channel population demand, leverage the proactive role of primary healthcare institutions, optimize medical resource allocation and treatment sequencing, enhance basic medical service accessibility, and effectively address the challenges of “difficulty in accessing medical care” and “high medical costs” (7). Despite years of reform efforts, the healthcare system of China remains plagued by persistent challenges of accessibility and affordability (8). While the hierarchical medical system holds immense potential to advance the universality and equity of medical services and modernize primary healthcare governance, questions persist regarding its effectiveness. Has the hierarchical medical system truly fulfilled its intended role? How efficacious is it in enhancing population health amid the current stage of development? Existing studies have failed to provide definitive answers to these critical questions.

“Strengthening the grassroots” has long been the focal point of China’s healthcare reform. The core tenets of the hierarchical medical system include “primary diagnosis and treatment, emergency and slow treatment, two-way referrals, and vertical integration” (9, 10). Technically, the hierarchical medical system enhances public access to high-quality medical services, whereas from a strategic standpoint, it serves as an effective avenue for overcoming challenges within the medical and healthcare system reform (11). Undoubtedly, the hierarchical medical system has been central to China’s healthcare reform efforts for years, playing a pivotal role in the comprehensive realignment of healthcare resources, mitigating supply–demand imbalances, shaping a novel model for healthcare reform, and addressing significant barriers to public healthcare access (12). From policy formulation to implementation at various levels, China has largely established a hierarchical diagnosis and treatment promotion model centered around medical service integration, supplemented by peer support, telemedicine, a family doctor contracting system, and health insurance reform (13–15). However, as China transitions into a new phase of development, a pertinent question arises: has the implementation of the hierarchical medical system genuinely enhanced the health of the population? The answer to this query not only impacts the smooth modernization of China’s primary healthcare governance system and capacity but also holds crucial theoretical implications for future healthcare system reforms. Thus, within the broader framework of promoting the Healthy China strategy, this study empirically evaluates the health implications of the hierarchical medical system. This endeavor not only provides novel empirical evidence for the evolution of China’s healthcare service system and population strategy in the new era but also provides theoretical guidance for advancing the comprehensive implementation of the Healthy China strategy and fostering the modernization of healthcare service capacity and systems. Such an endeavor bears significant theoretical and practical significance.

Based on this premise, this paper utilizes the latest 2020 China Family Panel Studies (CFPS) database to empirically analyze the effects of implementing the hierarchical medical system on population health. The notable contributions of this paper are: first, we employed the most recent 2020 CFPS data to empirically scrutinize the health impacts of implementing the hierarchical medical system in the new situation, thereby providing a theoretical foundation for formulating healthcare policies in alignment with the evolving social development stage in China; second, we conducted a comprehensive analysis of the health effects of the hierarchical medical system implementation by exploring sample heterogeneity characteristics, specifically focusing on healthcare insurance participation status and literacy levels; and third, we conducted an endogeneity test using the propensity score matching (PSM) model to rectify estimation errors arising from sample self-selection. The paper is organized as follows in the sections that follow: the second section entails a literature review; the third section delineates the design of the study, including data sources, variable settings, and model construction; the fourth section concentrates on result interpretation, encompassing baseline regression data and the outcomes of robustness tests; the fifth section engages in an in-depth discussion, incorporating findings from heterogeneity analysis and endogeneity tests; and the final section encapsulates the conclusions of the paper along with recommendations derived therein.

## 2 Literature review

The Eighth International Conference on Health Promotion saw the introduction of the “Health in All Policies” concept by the World Health Organization, which emphasized the necessity for nations to take the health consequences of policymaking processes into account (16, 17). “Integrating health into all policies” constitutes a significant aspect of China’s health strategy, serving as a pivotal approach to promoting the construction of a “Healthy China” and achieving health equity for all (18). Since the inception of the healthcare reform in 2009, numerous scholars have conducted extensive theoretical and empirical research on the efficacy of healthcare reform initiatives and policy enhancements. In recent years, with the progressive reform of China’s healthcare system, scholarly attention has shifted toward investigating the correlation between healthcare reform outcomes and population health. In the context of healthcare reform, Wang and Liu conducted an in-depth analysis of how the integration of urban and rural health insurance schemes affects the physical well-being of older adults in rural China, alongside mitigating health disparities. Their research indicated a significant improvement in the physical and mental health status of rural older adults following such integration, consequently reducing disparities in their health outcomes (19). The influence of major medical insurance programs on health outcomes and medical service utilization among urban and rural inhabitants was investigated, and the working mechanisms of the programs were explained, in Xu and Gu’s (20) study. Their findings revealed the efficacy of major illness insurance schemes in promoting inpatient medical services and enhancing health status among middle-aged and older adults, with medical service utilization playing a key role in this process (20). Regarding healthcare reform strategies, Feng et al. (21) conducted a systematic evaluation of medical service integration policies utilizing urban patient access data and hospital-level information. Their study revealed a substantial increase in outpatient visits and hospitalizations at secondary and tertiary hospitals following the implementation of medical service integration policies. Moreover, there was a notable surge in hospitalizations for minor chronic conditions at lower-level hospitals, indicating a shift toward decentralized healthcare delivery (21). Li et al. (22) analyzed monthly patient data from a community hospital in Beijing to assess the impact of contracting with community family physicians on the quality and efficiency of medical services received by older adult patients. Their findings demonstrated that contracting with community family physicians significantly reduced medical expenses and healthcare utilization frequency among the older adult, thereby promoting the hierarchical medical system and optimizing medical resource allocation (22). In the context of pharmaceutical reform, recent studies have investigated the economic benefits of modifications to the health insurance drug catalog on public health outcomes and medical expenses. These studies revealed a significant enhancement in patients’ health outcomes resulting from dynamic changes in the health insurance drug catalog (23).

As a pivotal policy design within China’s medical and healthcare system reform, the foundational objective of the hierarchical medical system is to facilitate the rational allocation and progressive decentralization of medical resources. This aims to alleviate longstanding challenges in China, namely, difficulties in accessing and affording healthcare services. The objective of these initiatives is to achieve the strategic vision of a Healthy China and to improve population health on a broad scale. The concept of hierarchical diagnosis and treatment originated in developed countries such as the United Kingdom and the United States, often referred to as “integrated healthcare” in global discourse (24, 25). Since China’s initiation of the hierarchical medical system in 2015, numerous scholars have focused on various aspects of this system, ranging from policy design and refinement to policy evaluation (26–28). In terms of theoretical research, Shen and Du (12) argue that implementing the hierarchical medical system is a strategic imperative for advancing China’s medical and healthcare system reform and plays a pivotal role in determining the success of such reforms. However, despite notable achievements in recent years, challenges persist, including significant resistance, slow progress, and difficulties in enhancing primary medical services (12). Dang and Li (29), drawing from historical institutionalism, contend that current difficulties in promoting the hierarchical medical system in China stem from mismatches between government-led institutional arrangements and the operational needs of healthcare institutions and community members, leading to systemic disorder. Wang systematically describes the motivation, policy process, model, content, effectiveness, and challenges of establishing China’s integrated healthcare service system. Through the “Conditional Mechanism Model of Healthcare Organizational Integration,” Wang and Wang (30) elucidates the theory and mechanism underlying healthcare organizational integration, offering theoretical innovations, reform pathways, and insights from Chinese experiences for the governance of healthcare service systems and the high-quality development of public organizations internationally. In terms of empirical research, Pan and Yang (31) empirically examine the impact of the hierarchical medical system on reducing health disparities in the older adult population and its operational mechanisms using data from the 2011–2018 Chinese Longitudinal Healthy Longevity Survey. Their findings highlight the system’s effectiveness in mitigating health disparities among the older adult (31). Wang (32) investigates the role of primary first-time medical care in residents’ health using self-examination data from residents of Hunan Province, revealing a positive effect of primary first-time medical care on residents’ health outcomes. Moreover, other scholars have conducted relevant empirical studies examining family financial burdens (33), patient satisfaction, and consultation behavior, as well as healthcare resource allocation (34).

The aforementioned studies have conducted systematic and in-depth analyses of the hierarchical medical system and its implications for population health, yielding a wealth of research findings. In terms of population health, researchers have predominantly explored the interplay between healthcare, health insurance, and pharmaceutical reforms. Regarding the hierarchical medical system, studies have primarily focused on evaluating policy effectiveness, refining policy design, or examining causal relationships between the policy and variables such as family financial burdens and patient healthcare behaviors. Nonetheless, there is still a dearth of research on the connection between population health and the hierarchical medical system, particularly as China enters a new developmental stage, characterized by population aging and pediatric healthcare needs. Amid initiatives to modernize the Chinese-style medical and healthcare governance system and progress the creation of a Healthy China, using the latest data for analyzing the health effects of the hierarchical medical system is pivotal. In order to strengthen the effects of healthcare system reforms and encourage the superior growth of medical and healthcare services, such initiatives are highly practical. To address this gap, this paper empirically investigates the impact of implementing the hierarchical medical system on population health using the latest 2020 CFPS. We have examined the system’s heterogeneity, robustness, and endogeneity, aiming to furnish empirical evidence to inform healthcare policy formulation in the new era. Through this endeavor, we seek to contribute to the enhancement of healthcare policy-making and the advancement of high-quality development in the healthcare sector.

## 3 Study design

### 3.1 Data sources

The 2020 CFPS database, created in 2010 and run by Peking University’s China Social Science Survey Center, provided the data used in this work. This poll has a large and highly representative sample size and covers 25 provinces, municipalities, and autonomous regions in the country. Given the thematic focus on hierarchical diagnosis and treatment and population health, the research sample was chosen using the adult questionnaire. The 2020 CFPS database was released in 2021 by the China Centre for Social Science Survey of Peking University. Within the 2020 CFPS data, the total number of adult samples amounted to 28,590. To ensure data integrity and reliability, missing values, outliers, and invalid variables were processed, selection criteria were applied, and ultimately, 10,970 usable specimens were acquired.

### 3.2 Variable design

#### 3.2.1 Dependent variable

The dependent variable in this paper is health status. While previous studies employed diverse health measurement standards, the use of self-assessed health as a measure has garnered widespread acceptance due to its validity and credibility. Building upon comprehensive previous research (35, 36) we used the status of chronic diseases and subjective self-assessed health as indices. Self-assessed health is derived from question P201: “How do you think your health is?” The responses are categorized into five levels: very healthy, relatively healthy, average, unhealthy, and very unhealthy, with scores ranging from 1 to 5, where higher scores denote poorer health. Chronic disease status was determined through question P401: “In the past 6 months, have you suffered from a chronic disease illness diagnosed by a doctor?” The responses were binary, with 1 indicating a positive diagnosis (i.e., unhealthy) and 2 indicating a negative diagnosis (i.e., healthy).

#### 3.2.2 Independent variable

The core tenet of the hierarchical medical system, as elucidated by the National Health Commission, revolves around “primary first diagnosis, “which serves as a pivotal aspect of the system. Thus, this paper selects primary first diagnosis behavior as the key variable to measure the impact of the hierarchical medical system (33). Within the 2020 CFPS database, question P601 addresses the usual location individuals seek medical attention. The hierarchical medical system is adhered to by respondents who frequently visit clinics, community health service stations, township health facilities, or community health service centers. These respondents are awarded a value of 1. Conversely, those who patronize general hospitals and specialist hospitals are assigned a value of 0, signifying non-adherence to the hierarchical medical system. Control Variables: In selecting individual parameters, variables such as sex, age, education level, marital status, type of residence, and medical insurance participation were primarily considered. In addition, recognizing the significant influence of daily living habits on individual health, variables including smoking, drinking, and physical activity were integrated into the model. The assignment and descriptive analysis of variables are presented in Table 1.

**Table 1:** Variable definitions and descriptive statistics.

Variables	Definitions	*N*	*M*	SD
Dependent variables				
Self-assessed health	Very healthy = 1; Very healthy = 2; Relatively healthy = 3; Average = 4, Unhealthy = 5	10,970	2.572	1.032
Chronic disease conditions	Not sick = 0; Sick = 1	10,970	0.061	0.238
Independent variables				
Hierarchical medical system	No = 0; Yes = 1	10,970	0.549	0.498
Control variables				
Sex	Female = 0; Male = 1	10,970	0.498	0.500
Age	Unit: years	10,970	30.983	8.351
Type of account	Agricultural = 1; Non-agricultural = 2	10,970	1.276	0.447
Marital status	Unmarried = 1; Married = 2; Cohabiting = 3; Divorced = 4; Widowed = 5	10,970	1.753	0.635
Educational level	Illiteracy = 1; Primary = 2; Secondary = 3; University = 4; Postgraduate = 5	10,970	2.342	0.582
Medical insurance	No = 0; Yes = 1	10,970	0.850	0.357
Frequency of physical exercise	Low = 1; Average = 2; High = 3	10,970	1.285	0.635
Smoking	No = 0; Yes = 1	10,970	0.258	0.438
Drinking	No = 0; Yes = 1	10,970	0.096	0.294

### 3.3 Model building

Several econometric models were used to examine the relationship between the hierarchical medical and population health since the explanatory factors for population health status in this study are both binary and five-categorical. For the five-categorical variables, we adopted the following Ordered Probit regression model (37):


(1) $${\text{Health}}_{i}^{*}=\alpha +\beta {\text{Healthcare}}_{i}+\gamma Z_{i}+\varepsilon_{i}$$

In Eq. 1, ${\text{Health}}_{i}^{*}$ denotes the latent variable of the population health level, ${\text{Healthcare}}_{i}$ denotes the hierarchical medical system, $Z_{i}$ represents the control variable affecting health status, and $\varepsilon_{i}$ represents the random disturbance term. For binary categorical variables, we employed the following Probit regression model:


(2) $$Pr\left(Y_{i}=1\right)=\phi \left(\alpha +\beta {\text{Healthcare}}_{i}+\gamma Z_{i}+\varepsilon_{i}\right)$$

In Eq. 2, i denotes an individual, $Y_{i}$ indicates the health of the individual i, ${\text{Healthcare}}_{i}$ represents the hierarchical medical system, $Z_{i}$ represents the control variable, $\mu_{i}$ indicates the random interference term, and β and γ represent the coefficients of the variables.

Individual decisions to respond positively to the hierarchical medical system and opt for primary care are influenced by several factors, including age, type of occupation, and education level. These choices are based on unique situations and could cause sample selectivity bias and endogeneity issues. We used a PSM model to quantify the overall impact of the hierarchical medical system on population health in order to address this problem (38, 39). To lessen interference and the impact of external factors, the PSM model splits the sample into treatment and control groups and phases the analysis based on propensity score. The following is how the model was created:


(3) $$Z_{i}=Z_{0i}+\left(Z_{1i}-Z_{0i}\right)G_{i}$$

(4) $$ATT=E\left(Z_{1i}-Z_{0i}|G_{i}=1\right)$$

In Eq. 3, $G_{i}$ denotes the treatment variable, taking the value of 1 if individual i is in the experimental group (implementing hierarchical medical system), and 0 if individual i is in the treatment group (not implementing hierarchical medical system). The two clusters—the treatment group, which implemented the hierarchical medical system, and the control group, which did not—are the main explanatory variables in this study. The average treatment effect of the treatment group, or the overall impact of the hierarchical medical system on population health, is represented by Eq. 4.

## 4 Results

### 4.1 Results of baseline regression

The results in Table 2 indicate significant effects of the hierarchical medical system on population health at the 1% level of significance, demonstrating its efficacy in improving overall health. In model (a), without the inclusion of control variables, the hierarchical medical system improves individuals’ self-assessed health status by 0.114 units in a positive direction. This effect slightly diminishes to 0.098 units after controlling for personal characteristics and lifestyle factors. Model (c) reveals that the probability of the hierarchical medical system contributing to an improvement in chronic disease status is 39.7% without control variables, increasing to 42.9% when control variables are included. These results indicate the significant enhancement of population health facilitated by the hierarchical medical system. The analysis of control variables aligns with expectations. Age exhibits a negative correlation with self-assessed health status and an increase in the prevalence of chronic diseases. Higher literacy levels correlate with better self-assessed health, as more educated individuals tend to opt for primary care and utilize available resources to improve health. Additionally, greater participation in physical activity correlates with better self-rated health, while the effect on chronic disease status is not significant.

**Table 2:** Results of the empirical analysis of the impact of the hierarchical medical system on population health.

Variables	Model (a)	Model (b)	Model (c)	Model (d)
	Self-assessed health	Self-assessed health	Chronic disease conditions	Chronic disease conditions
Hierarchical medical system	−0.114^***^ (0.021)	−0.098^***^ (0.022)	−0.397^***^ (0.039)	−0.429^***^ (0.041)
Sex		−0.131^***^ (0.025)		−0.030 (0.049)
Age		0.029^***^ (0.002)		0.028^***^ (0.003)
Type of account		0.047^*^ (0.024)		−0.028 (0.046)
Marital status		0.013 (0.020)		0.026 (0.036)
Educational level		0.067^***^ (0.018)		0.006 (0.034)
Medical insurance		0.016 (0.030)		0.118^*^ (0.063)
Frequency of physical exercise		−0.054^***^ (0.016)		0.025 (0.031)
Smoking		−0.023 (0.029)		0.080 (0.005)
Drinking		−0.061 (0.037)		−0.079 (0.069)
*N*	10,970	10,970	10,970	10,970
Adj-X2	0.0011	0.0237	0.0213	0.0558

^*^, ^**^, and ^***^ represent significance at 10, 5, and 1%, respectively.

### 4.2 Robustness evaluations

To further analyze the impact of the hierarchical medical system on population health, robustness testing employing replacement estimation models was conducted. Given the five-categorical and two-categorical variables in this study, OLogit and Logit models were utilized to assess the relationship between the hierarchical medical system and population health indicators. Consistent with baseline regression findings, the estimation results of models (e) and (f) show a substantial negative association at the 1% level of significance, suggesting that the hierarchical medical system efficiently enhances the population’s self-assessed health status. Similarly, the estimation results of models (g) and (h) demonstrate significant negative correlations at the 1% level of significance, signifying the hierarchical medical system’s substantial improvement of the population’s chronic disease status. Overall, these robustness tests affirm the reliability and consistency of the estimation results. Table 3 presents the results of the robustness test.

**Table 3:** Robustness test results.

Variable	Model (e)	Model (f)	Model (g)	Model (h)
	Self-assessed health	Self-assessed health	Chronic disease conditions	Chronic disease conditions
Hierarchical medical system	−0.199^***^ (0.036)	−0.160^***^ (0.037)	−0.841^***^ (0.083)	−0.884^***^ (0.088)
Sex		−0.239^***^ (0.044)		−0.058 (0.103)
Age		0.052^***^ (0.003)		0.056^***^ (0.005)
Type of account		0.019 (0.035)		0.062 (0.072)
Marital status		0.114^***^ (0.033)		0.016 (0.071)
Educational level		0.094^**^ (0.042)		−0.073 (0.094)
Medical insurance		0.025 (0.051)		0.229^*^ (0.136)
Frequency of physical exercise		−0.098^***^ (0.028)		0.058 (0.063)
Smoking		−0.048 (0.051)		0.147 (0.114)
Drinking		−0.094 (0.066)		−0.169 (0.144)
*N*	10,970	10,970	10,970	10,970
Adj-X2	0.0011	0.0246	0.0213	0.0547

^*^, ^**^, and ^***^ represent significance at 10, 5, and 1%, respectively.

## 5 Further analysis

### 5.1 Heterogeneity analysis

Given the influence of individual endowment resources, the impact of the hierarchical medical system on the health status of different groups may exhibit significant heterogeneity. To delve deeper into the variability of this impact, we conducted a heterogeneity test from the perspectives of health insurance participation and different literacy levels. The findings presented in Table 4 indicate that the health status of all groups with health insurance is more significantly impacted by the hierarchical medical system. In particular, it improves the chronic illness status and self-rated health of people with health insurance quite well. Conversely, for those not participating in health insurance, the hierarchical medical system primarily improves chronic disease status, with insignificant effects on self-assessed health. Furthermore, the group with secondary school education and below is more significantly impacted by the hierarchical medical system in terms of health than is the group with university degree and above. These results show that the impact of the hierarchical medical system on population health status varies significantly depending on literacy and health insurance participation levels. Table 4 displays the heterogeneity test findings.

**Table 4:** Results of heterogeneity analysis.

Variables	Medical insurance				Level of education			
	Non-participants		Participants		Secondary and below		University and above	
	Self-assessed health	Chronic disease conditions	Self-assessed health	Chronic disease conditions	Self-assessed health	Chronic disease conditions	Self-assessed health	Chronic disease conditions
Hierarchical medical system	−0.077 (0.055)	−0.551^***^ (0.129)	−0101^***^ (0.023)	−0.415^***^ (0.044)	−0.109^***^ (0.021)	−0.436^***^ (0.041)	−0.093 (0.211)	−0.013 (0.364)
Control variables	Yes	Yes	Yes	Yes	Yes	Yes	Yes	Yes
Adj-R2	0.0220	0.1264	0.0232	0.0468	0.0232	0.0571	0.0394	0.0573

### 5.2 Endogenous processing

The choice of residents of primary care is an independent decision influenced by factors such as sex, household registration, and education, leading to sample selectivity bias and potentially inaccurate model estimation results. We used the PSM model to evaluate the net effect of the tiered diagnostic and treatment policy on population health status in order to reduce sample selectivity bias. To estimate the outcomes, four procedures were used: K-nearest-neighbor matching, K-nearest-neighbor caliper matching, and others (40, 41). A balance check was conducted to ensure the stability of the matching results. The matching effect was deemed to be weak and the prediction findings may be skewed toward invalidity if there was a clear difference between the two sample groups following matching, and vice versa. Due to spatial constraints, we have just presented the outcomes of the self-assessed health balancing test (Table 5).

**Table 5:** Sample matching quality balance test.

Variable	Unmatched matched	Mean		Bis (%)	Reduce Bias (%)	*T*-test	
		Treated	Control			*t*	*p* > |t|
Sex	U	0.505	0.489	3.2	85.0	1.65	0.099
	M	0.505	0.507	−0.5		−0.26	0.795
Age	U	31.038	30.917	1.5	48.0	0.75	0.451
	M	31.016	30.953	0.8		0.41	0.679
Type of account	U	1.759	1.746	2.0	42.6	1.07	0.286
	M	1.758	1.766	−1.2		−0.64	0.521
Marital status	U	2.245	2.460	−37.6	97.7	−19.61	0.000
	M	2.246	2.251	−0.9		−0.48	0.632
Educational level	U	1.180	1.391	−48.1	98.4	−25.34	0.000
	M	1.180	1.177	0.8		0.50	0.618
Medical insurance	U	0.844	0.858	−3.9	47.5	−2.02	0.043
	M	0.844	0.852	−2.0		−1.11	0.266
Physical exercise	U	1.252	1.324	−11.3	99.6	−5.94	0.000
	M	1.252	1.253	−0.1		−0.02	0.980
Smoking	U	0.270	0.244	6.0	89.3	3.12	0.002
	M	0.270	0.267	0.6		0.35	0.729
Drinking	U	0.099	0.091	2.8	66.5	1.44	0.151
	M	0.099	0.102	−0.9		−0.50	0.620

Treated denotes the treatment group and Control denotes the control group.

**Figure 1:**
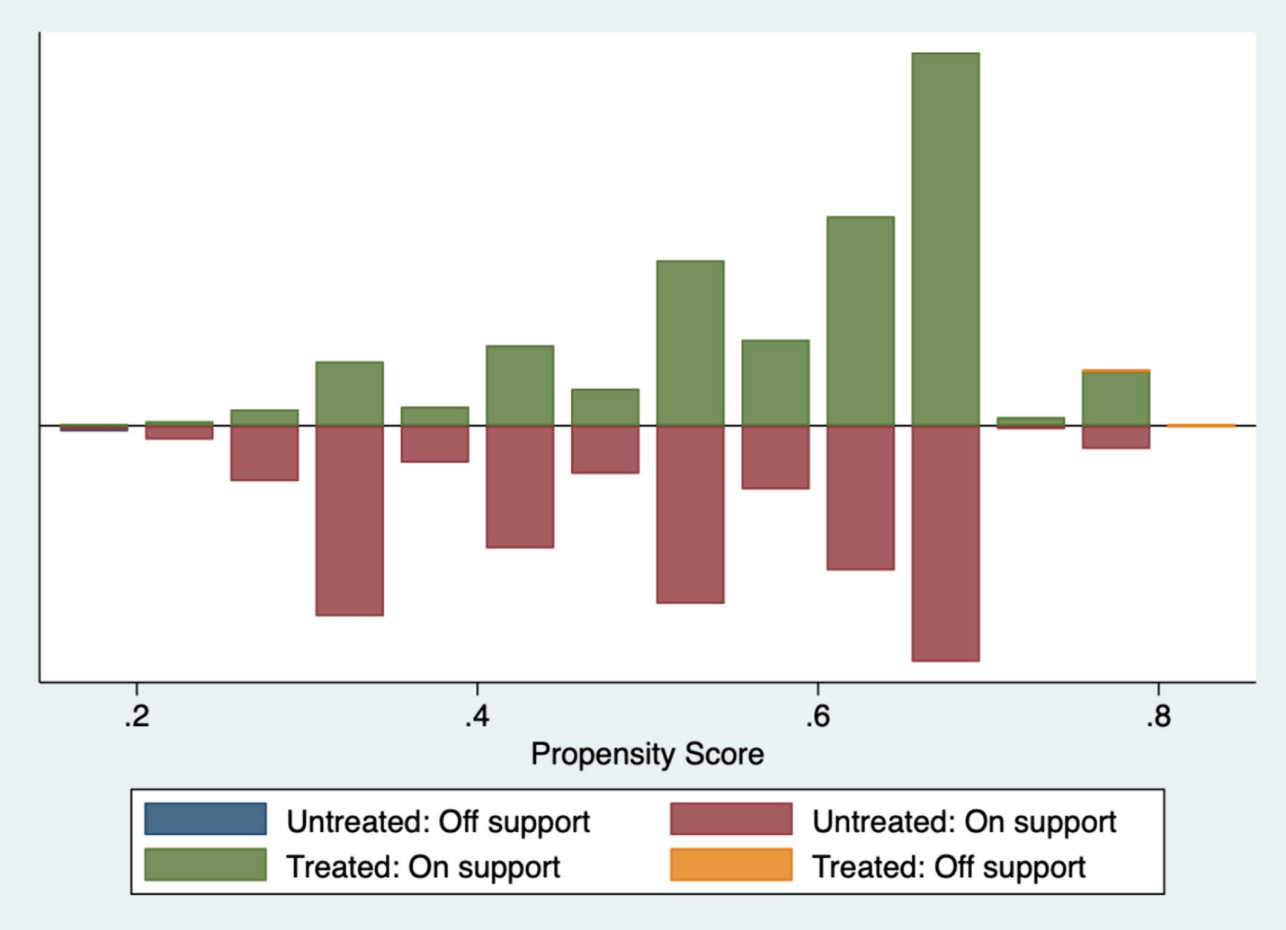
Distribution of sample matches.

**Figure 2:**
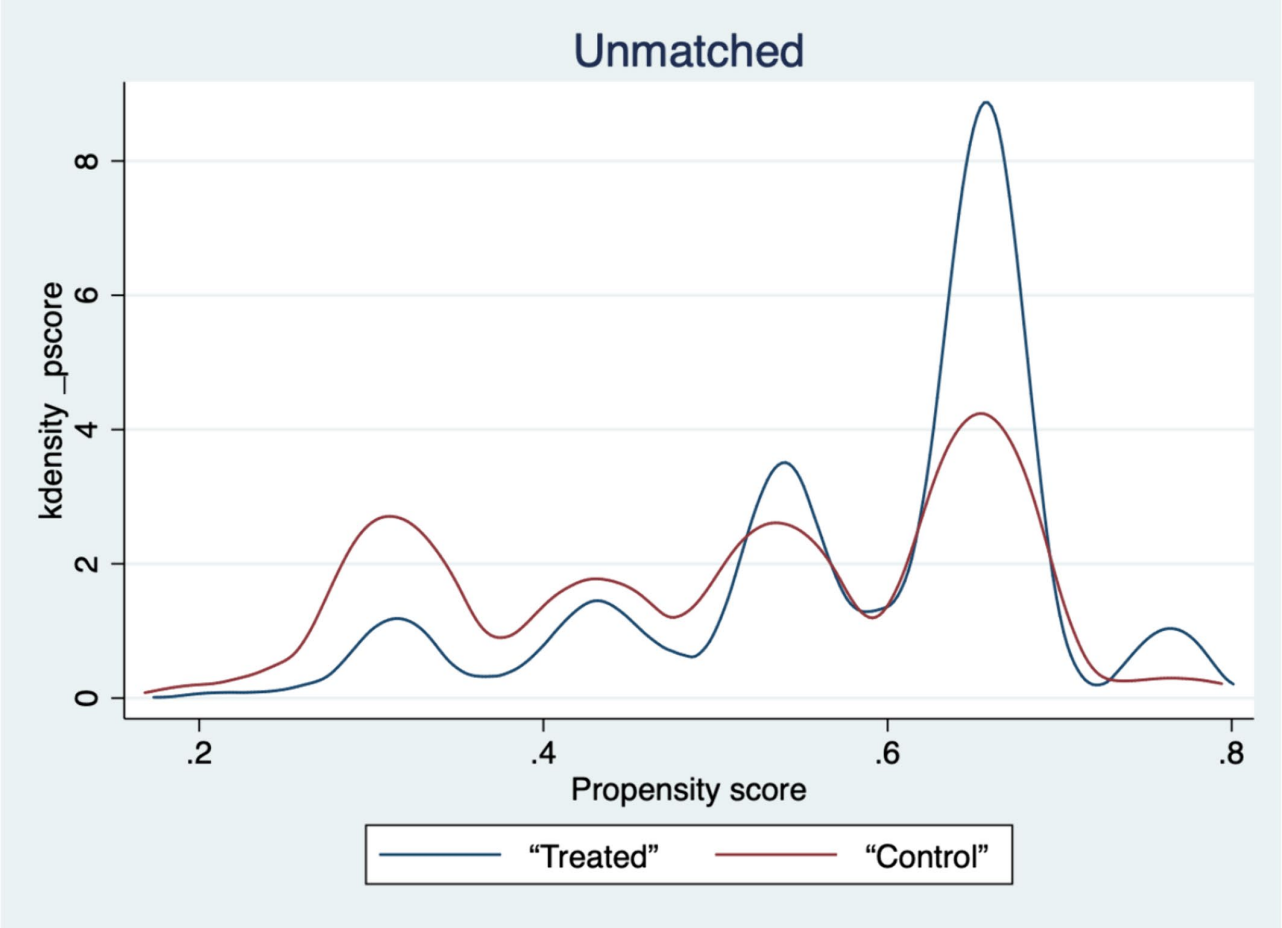
Plot of kernel density function before matching.

**Figure 3:**
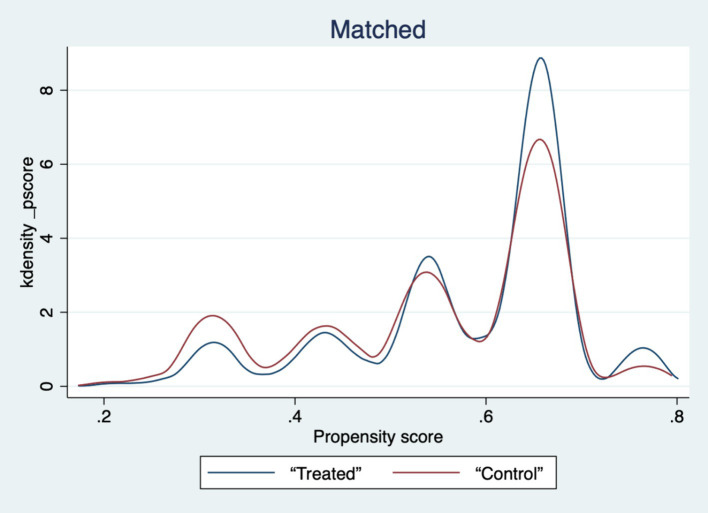
Plot of kernel density function after matching.

Table 5‘s findings show that, for every variable, the standardized deviation’s absolute value following matching is less than 5%. The *t*-values of the variables in the treatment and control groups are significant before matching but not after matching, with the exception of a small number of variables, according to mean *t*-tests. Plots of the sample matching distribution (see Figure 1) and the kernel density function before and after matching (see Figures 2, 3) show that the matching distribution smoothes out and that the treatment and control groups’ curves become more similar and eventually converge after matching. These findings imply that following matching, there is no longer a substantial difference between the treatment and control groups, improving matching outcomes and solving the endogeneity issue brought on by sample selection bias.

The average treatment effect (ATT) of the impact of the hierarchical medical system on the health of the population is shown in Table 6. Before matching, the ATT values for the population’s self-assessed health and chronic disease status were − 0.111 and − 0.047, respectively. After employing the K-nearest neighbor matching strategy, these values changed to −0.083 and − 0.057, indicating a slight reduction in magnitude. Upon controlling for sample selectivity error, the net effect of individual self-assessed health status and chronic disease impacts of the tiered diagnosis and treatment policy were 8.3 and 5.7%, respectively. K-nearest neighbor matching yields results that are corroborated by radius neighbor matching, kernel matching, and caliper matching. The net effect of the hierarchical medical system on the population’s self-assessed health is 8.6, 9.0, and 9.1%, whereas the net effect on chronic disease status is 5.7, 5.0, and 4.9% using K-nearest neighbor caliper matching, radius neighbor matching, and kernel matching, respectively. This consistency across different matching methods underscores the stability of the results obtained using PSM. These findings emphasize the importance of eliminating sample selection bias, as failure to do so may lead to overestimation of the hierarchical medical system’s impact on individual self-assessed health while disregarding its positive effect on alleviating chronic disease problems.

**Table 6:** Results of propensity score matching estimation.

	Self-assessed health				Chronic disease conditions			
	Treated	Control	ATT	SE	Treated	Control	ATT	SE
Unmatched	2.522	2.632	−0.111	0.020	0.039	0.086	−0.047	0.005
Matched								
K-nearest neighbor matching	2.521	2.604	−0.083	0.024	0.039	0.096	−0.057	0.006
K-nearest neighbor caliper matching	2.521	2.607	−0.086	0.024	0.039	0.096	−0.057	0.006
Radius-neighbor matching	2.521	2.611	−0.090	0.021	0.039	0.089	−0.050	0.005
Kernel matching	2.521	2.612	−0.091	0.021	0.039	0.089	−0.049	0.005

## 6 Discussion

### 6.1 General findings

We empirically examined the impact of the implementation of the hierarchical medical system on the population’s health level using the latest 2020 CFPS database and explored the variability of this impact across different health insurance participation statuses and different literacy groups. The results of this study indicate that the implementation of the hierarchical medical system has significantly improved population health in China, fulfilling the original purpose of the policy design. The success of the hierarchical medical system can be attributed to several factors. First, it promotes the downward sinking of high-quality medical resources, thereby enhancing the accessibility of primary medical services. Second, it prevents the wastage of medical resources and enables their rational and effective allocation. President Xi Jinping pointed out that “Health is the most important indicator of a happy life; health is 1, and the others are the zeros that follow; without 1, more zeros are meaningless.” The CPC and Chinese government have consistently prioritized people’s health and wellness. Since the 18th National Congress of the CPC, people’s health has been positioned for priority development, signifying its critical role in national prosperity and wealth. The findings of the heterogeneity analysis demonstrate how the hierarchical medical system significantly con-tributes to the improvement of the self-assessed health and chronic disease status of those enrolled in health insurance, underscoring the institution’s critical role in China’s healthcare system. Additionally, we used the PSM model to estimate the net effect of the tiered diagnostic and treatment policy on the population’s health condition in order to solve the sample self-selection issue. The outcomes show how well the PSM model handles the endogeneity issue.

### 6.2 Policy implications

We present the following policy recommendations: first is the continued promotion of the hierarchical medical system. Undoubtedly, the hierarchical medical system has played a significant role in redistributing medical and health resources and improving primary healthcare in China. The findings of this paper also demonstrate its positive impact on both the self-assessment of population health and the management of chronic diseases. Therefore, amidst the comprehensive promotion of the Healthy China strategy and the deepening of healthcare reform, it is imperative to steadfastly promote the implementation of the hierarchical medical system. This will enable it to further contribute to the modernization of the national healthcare system. Second, the implementation of the hierarchical medical system should be comprehensively promoted. The study reveals significant differences in the effects of the hierarchical medical system on the health outcomes of different demographic groups. Concurrently, it identifies various challenges in policy implementation across different regions and governmental levels, such as in-adequate execution and failure to effectively implement policies. Therefore, there is a pressing need to comprehensively promote the implementation of the hierarchical medical system. This includes improving policy monitoring and evaluation mechanisms to ensure that reform efforts yield tangible benefits for grassroots communities. Third, the hierarchical medical system should be actively used to upgrade the health of the population. The hierarchical medical system can promote individual health through several avenues, including the family doctor system, health insurance payment catalog, and establishment of health records. Therefore, it is necessary to leverage the various policy tools in the hierarchical medical system to comprehensively improve population health. Incentives for hospitals and healthcare personnel can be further increased, and a competitive remuneration system should be formulated to facilitate the equitable distribution of high-quality medical resources and prevent resource wastage. Moreover, the family doctor system should be further improved to optimize the role of family doctors in disease diagnosis, treatment, and preventive healthcare. Encouraging individuals to prioritize preventive healthcare can mitigate difficulties and high costs associated with accessing medical care at the primary level. Fourth, it is necessary to pay attention to the impact of regional differences and the heterogeneity of population classes. On the one hand, due to the large differences in the level of economic development between regions in China, there will be significant differences in the implementation of the hierarchical medical care policy in different regions on the promotion of the health of the population, and at the same time the differences between urban and rural areas will also have a certain impact on the results. On the other hand, the degree of implementation of the hierarchical medical care policy varies greatly among different groups, which will have different impacts on the health promotion effect of analyzing medical care. Therefore, government departments should pay attention to the differences between the geographical areas and the grassroots level of the population in the future policy formulation, and formulate health promotion policies according to the local conditions.

### 6.3 Innovations and shortcomings

This paper focuses on the relationship between healthcare system design and population health to provide empirical evidence for the design of China’s healthcare system in the new era. The innovation of this paper lies in the following: first, we focused for the first time on the topic of the improvement of population health by the hierarchical medical system, and empirically analyzed the causal relationship between the impact of the hierarchical medical system on population health, providing important evidence for the continuation and enhancement of the hierarchical medical system in China’s new era. Second, we utilized the newly released CFPS database to conduct analyses, which is a significant reference and guide for healthcare system reform in the new era. Third, in order to address the sample selectivity bias-related endogeneity issue, we used the PSM model to eliminate endogeneity, thereby obtaining the net effect of the hierarchical medical system on population health. However, there are some shortcomings in this paper. Firstly, although the chosen control variables in this study include many aspects, there are inevitably omissions that may impact the estimation results. For example, as the Internet continues to grow in popularity, Internet use may also have an impact on residents’ implementation of hierarchical care. Residents who use the Internet may have a stronger willingness to implement hierarchical health care than those who do not, which in turn promotes their health more. Then again, urban–rural differences also have an impact on the willingness to receive hierarchical medical care. Residents living in large cities tend to have higher access to medical services than those in rural areas, and therefore have a higher awareness of hierarchical medical care, and therefore a higher health status than those living in rural areas. In addition, due to data availability, the measurement of tiered care in this paper is based on the measurement of the perspective of primary care, which, although it is a core component of tiered care, inevitably neglects other key elements of the healthcare system. This perspective may limit the results of the analysis and may not fully reflect the overall impact of the healthcare system on population health. This is one of the limitations of this paper. Addressing these issues will guide future research directions.

## 7 Conclusion

The foundation of national economic and social progress is health. The hierarchical medical system, as a policy framework intended to improve population health, is of enormous practical significance for advancing the Healthy China agenda and increasing the overall health of China’s population. Using the most recent 2020 CFPS information, we conducted an empirical investigation of the effects of the hierarchical medical system’s implementation on the health of the population. Additionally, we investigated the variations in these effects among various health insurance enrollment statuses and literacy groups. The following are the article’s primary conclusions: First, China’s population is much healthier now that the hierarchical medical system has been put into place. This is evident in the improvement of self-assessed health as well as the notable reduction in chronic diseases, which is sustained even after testing with the replacement estimation model. Second, there is a great deal of variation in the impact of the hierarchical medical system on each person’s health state with regard to health insurance participation and literacy. In particular, with regard to health insurance enrollment, the hierarchical medical system primarily improves the status of chronic diseases and self-assessed health for those who participate in it, while having little effect on self-assessed health for those who do not enroll. In terms of literacy, the group with secondary school education and less is more significantly impacted by the hierarchical medical system than is the group with university education and above in terms of health. Third, we used PSM to address the sample selectivity bias endogeneity issue. The outcomes showed that the PSM model successfully reduces endogeneity. The findings also show that if sample selectivity bias is not eliminated, the impact of the hierarchical medical system on self-assessed health may be overestimated, while its influence on chronic illness conditions may be underestimated.

## Data availability statement

The original contributions presented in the study are included in the article/supplementary material, further inquiries can be directed to the corresponding author.

## Author contributions

YW: Conceptualization, Formal analysis, Funding acquisition, Investigation, Methodology, Project administration, Software, Writing – original draft, Writing – review & editing. HC: Conceptualization, Methodology, Supervision, Validation, Visualization, Writing – review & editing.
